# Influence of Vanillin at Different Concentrations on the Properties of Commercial Alginate Impression Materials: An *in-vitro* Study.

**DOI:** 10.12688/f1000research.184245.2

**Published:** 2026-10-06

**Authors:** Sai Surya Sameehan Adavi, Nayana Prabhu, Kishore Ginjupalli, Runki Saran, P Kalyan Chakravarthy, Disha Prabhu, Veena Hedge, Pradeep S

**Affiliations:** 1 Department of Prosthodontics and Crown & Bridge, Manipal College of Dental Sciences, Manipal Academy of Higher Education, Manipal, Karnataka, India; 2 Department of Prosthodontics and Crown & Bridge, Manipal College of Dental Sciences, Manipal Academy of Higher Education, Manipal, Karnataka, India; 3 Department of Dental Materials, Manipal College of Dental Sciences, Manipal Academy of Higher Education, Manipal, Karnataka, India; 4 Department of Dental Materials, Manipal College of Dental Sciences, Manipal Academy of Higher Education, Manipal, Karnataka, India; 5 Department of Public Health Dentistry, Manipal College of Dental Sciences, Manipal Academy of Higher Education, Manipal, Karnataka, India; 6 Department of Conservative Dentistry & Endodontics, Manipal College of Dental Sciences, Manipal Academy of Higher Education, Manipal, Karnataka, India; 7 Department of Prosthodontics and Crown & Bridge, Manipal College of Dental Sciences, Manipal Academy of Higher Education, Manipal, Karnataka, India; 8 Department of Prosthodontics and Crown & Bridge, Manipal College of Dental Sciences, Manipal Academy of Higher Education, Manipal, Karnataka, India

**Keywords:** Alginate impression material, Irreversible hydrocolloid, Vanillin, Gel strength, Gelation time, Permanent deformation, Flow, Tear strength

## Abstract

**Background:**

Alginate is a widely used irreversible hydrocolloid impression material in dentistry because of its ease of use, affordability, and acceptable clinical performance. Modification of alginate with additives may alter its physical and mechanical properties. This study evaluated the effect of incorporating vanillin at different concentrations into two alginate impression materials, Zelgan and Neocolloid.

**Methods:**

Vanillin was incorporated into Zelgan and Neocolloid alginate impression materials at concentrations of 0.5% and 1%. The materials were mixed with deionized water using a figure-of-8 motion for 45 seconds and moulded in polyvinyl chloride moulds to prepare specimens. Gel strength, permanent deformation, gelation time, flow, and tear strength were evaluated using standardized methods. Data were analyzed using the Shapiro–Wilk test for normality, followed by ANOVA or Kruskal–Wallis tests with appropriate post-hoc analyses. Between-group comparisons were performed using the independent-samples t-test or Mann–Whitney U test. Statistical significance was set at P < 0.05.

**Results:**

Two-way ANOVA showed significant main effects of alginate type and vanillin concentration on gel strength, gelation time, flow, and tear strength (all P<0.05), with Zelgan and Neocolloid differing significantly in gel strength, gelation time, and tear strength overall. A significant alginate × concentration interaction was found for gelation time and flow, indicating a material-dependent effect of vanillin on these properties, whereas gel strength and tear strength were affected by vanillin concentration similarly in both materials (no significant interaction). Permanent deformation was not significantly affected by alginate type, concentration, or their interaction. Tear strength was significantly greater at 1% vanillin than at control or 0.5% concentrations.

**Conclusions:**

Vanillin reduced gelation time and flow in both materials, with the magnitude of reduction differing significantly between Zelgan and Neocolloid. Vanillin also significantly increased tear strength at 1% concentration, an effect that did not differ significantly between the two materials. Vanillin may therefore be a useful additive for modifying selected properties of irreversible hydrocolloid impression materials.

## Introduction

Irreversible hydrocolloid impression materials, commonly known as alginates, are widely used for recording preliminary impressions of dentulous and partially dentulous mouths. It is supplied as a powder containing sodium alginate, calcium sulfate, trisodium phosphate, and fillers that form a gel upon mixing with water through a chemical reaction regulated by trisodium phosphate. ^1^ ^,^ ^2^ Alginate is known for its ability to record fine details due to its hydrophilic nature and good flow and is a cost-effective material with a relatively simple manipulation technique. However, its use is limited due to poor dimensional stability, lower tear strength and lack of antimicrobial action. Dimensional changes arise from imbibition, which occurs during conventional immersion or spray disinfection of impression and may compromise accuracy due to the material’s hydrophilic nature. ^3^

As alginate lacks antimicrobial activity, cross-infection between the clinic and the laboratory, or among patients, is a concern. To address this, self-disinfecting alginates incorporating antimicrobial agents have been developed. Various antimicrobial additives, including chlorhexidine, quaternary ammonium compounds such as didecyldimethyl ammonium chloride, fluoride, silver nanoparticles, magnesium oxide nanoparticles, and titanium dioxide nanoparticles, have been investigated for incorporation into impression materials to impart self-disinfecting properties and reduce the risk of cross-contamination. ^3^ ^–^ ^7^ The incorporation of antimicrobial additives alters the alginate-to-water ratio, directly affecting the properties of the resultant formulations. In this regard, it is pertinent to select an additive that imparts superior antimicrobial activity at lower concentrations without significantly altering the properties of alginate. ^8^ ^,^ ^9^

Vanillin (4-hydroxy-3-methoxybenzaldehyde), a naturally occurring phenolic compound derived from vanilla, exhibits broad-spectrum antimicrobial activity by disrupting bacterial cell membranes and inhibiting essential enzymes. ^10^ It has been investigated as a constituent of mouthrinses, as a reducing agent for the synthesis of antimicrobial silver nanoparticles. ^11^ The efficiency of Vanillin as an additive to impart antimicrobial properties to irreversible hydrocolloids was investigated earlier. However, addition of additives to gel matrix such as irreversible hydrocolloid impression material can significantly affect the clinically relevant characteristics such as gelation time, flow, gel strength, permanent deformation, tear strength etc. Hence, the present study investigated the effect of incorporation of vanillin (0.1%, 0.5%, and 1% w/w) into commercially available irreversible hydrocolloid impression materials on the aforementioned properties.

Leelapong et al. (2024) previously evaluated similar vanillin concentrations (0.1%, 0.5%, and 1% w/w) in a single irreversible hydrocolloid product, assessing working time, setting time, elastic recovery, and strain in compression under ISO 21563:2021. However, no study to date has directly compared two distinct commercial alginate formulations or examined their gelation time, gel strength, permanent deformation, and tear strength following incorporation of vanillin in various concentrations. The present in vitro study aimed to evaluate the physical properties of two commercially available irreversible hydrocolloid impression materials- Zelgan (Dentsply Sirona) and Neocolloid (Zhermack) – incorporated with vanillin at 0.5% and 1% concentrations. The present in vitro study aimed to evaluate the physical properties of two commercially available irreversible hydrocolloid impression materials- Zelgan (Dentsply Sirona) and Neocolloid (Zhermack) – incorporated with vanillin at 0.5% and 1% concentrations.

## Materials & methodology

Two commercially available irreversible hydrocolloid materials- Zelgan (Dentsply Sirona) and Neocolloid (Zhermack) were selected as shown in Table 1.

**Table 1.** Product name and manufacturer details.

Material used	Product information	Manufacture
Zelgan Impression material	Conventional alginate impression material Product batch number 423889	Dentsply Sirona Pvt, Ltd, India
Neocolloid Impression material	Conventional alginate impression material Product batch number 0000438835	Zhermack S.P.A, Italy

Vanillin (4-hydroxy-3-methoxybenzaldehyde) purity-99.5%, quantity- 100 gms was procured from Nano Research Lab, Jharkhand, India, Catalogue number-NRL0219042/215. Vanillin at 0.5% and 1% (w/w) concentration was incorporated into alginate impression materials. The sample size was estimated using G*power (v3.1.9.7). Based on an effect size of 0.63, with 80% power and 95% confidence, a minimum of 9 samples per group was required. Study design is depicted in Table 2.

**Table 2.** The prepared specimens were divided into various groups as described.

Group Name	Description
1A	Zelgan
1B	Zelgan +0.5% Vanillin
1C	Zelgan +1% Vanillin
2A	Neocolloid
2B	Neocolloid +0.5% Vanillin
2C	Neocolloid +1% Vanillin

### Specimen preparation

A precisely measured quantity of alginate impression material was blended with vanillin at either 0.5% or 1% by weight by transferring both into a container and mixing thoroughly to achieve homogeneous distribution. Unmodified alginate powder served as the control group. Test specimens were fabricated by combining a weighed quantity of alginate (with or without vanillin) with a measured volume of deionised water at room temperature (20°C) following the respective manufacturer's instructions (powder-to-water ratio: Zelgan 18g: 36 mL; Neocolloid 18g: 36 mL). The powder and water were placed into a rubber mixing bowl and spatulated for 45 seconds in a figure-of-8 motion by a single operator to minimise variability in manipulation. Mixing was continued until a smooth, homogeneous consistency was obtained. For specimen fabrication, the resulting mix was packed into a polyvinyl chloride (PVC) cylindrical mould (internal diameter: 30 mm; height: 16 mm) resting on a glass slab. A smaller secondary mould (internal diameter: 15 mm; height: 19 mm) was then inserted into the first mould until the material was expressed from the top, ensuring complete mould filling and eliminating air entrapment. A flat glass plate was subsequently pressed over the second mould to remove any excess material, and the assembly was left undisturbed for 5 minutes to allow complete gelation. Specimens were then carefully removed and subjected to testing. All specimens were fabricated in strict accordance with ADA Specification No. 18/ISO 1563. For each group, 1 independent mixtures were prepared, with 1 specimen obtained per mixture, such that each specimen constituted an independent experimental unit/was derived from a separate mixture. All testing instruments (universal testing machine, timing devices) were calibrated according to the manufacturer prior to use.

### Measurement of gelation time

Gelation time was assessed using a method adapted from Lemon et al. (2003). ^12^ Beginning 60 seconds after the initiation of mixing, a cylindrical polymethyl methacrylate (PMMA) rod (diameter: 6 mm; length: 10 cm) with a flat, polished tip was gently brought into contact with the surface of the alginate mix and lifted away. This contact-and-withdrawal procedure was carried out at 5-second intervals. The endpoint was identified as the moment when the material ceased to adhere to the rod tip. Gelation time was recorded as the total elapsed time from the commencement of mixing to this endpoint.

### Measurement of flow

Flow was assessed following the method outlined by Wang et al. (2007). ^13^ A 0.5 mL volume of freshly mixed alginate was dispensed onto a glass plate via a disposable syringe. A second glass plate was positioned over the material, and a 1 kg weight was applied to the upper plate for a duration of 5 seconds. The resulting disc of material was measured at three equidistant points, and the mean diameter (in mm) was recorded as the flow value.

### Measurement of gel strength

Compressive gel strength was evaluated using a technique adapted from MacPherson et al. (1967). ^14^ Six minutes after the start of mixing, specimens were removed from their moulds and positioned on the lower plate of a universal testing machine (Instron Model 3366, Instron Corporation, United Kingdom). A compressive load was applied at a crosshead speed of 10 mm/minute. The peak load recorded at the point of material failure — identified by a marked and sudden drop in the load–displacement curve — was taken as the gel strength value.

### Measurement of permanent deformation

Permanent deformation was determined using a previously established protocol with minor modifications. At 6 minutes from the start of mixing, each specimen was subjected to a compressive load sufficient to reduce its height by 10% of its original dimension. This deformation was sustained for 15 seconds, after which the load was released, and the specimen was allowed to recover freely for 30 seconds. The final height was then recorded, and permanent deformation was calculated as follows:

Permanent Deformation(%)=(Change in Length/Original Length)×100

### Measurement of tear strength

Tear strength specimens were prepared by packing freshly mixed alginate into a rectangular mould (70 × 25 × 1.5 mm). A cellophane sheet was placed over the mould surface to prevent adhesion, followed by a glass plate on top, upon which a load was applied to ensure complete and uniform mould filling. Once gelation was complete, specimens were carefully retrieved and trimmed of excess flash material using a surgical blade. Each specimen was then split longitudinally for 50 mm of its length, leaving the remaining 20 mm intact for gripping. The two resulting flanges were clamped into the tensile jigs of a universal testing machine (Instron Model 3366, UK), and a tensile force was applied at a rate of 50 mm/minute until complete separation occurred. Tear strength was calculated by dividing the maximum recorded load by the specimen thickness, and results were expressed in N/mm.

### Statistical analysis

Data were analysed using SPSS v26. Normality was assessed by the Shapiro-Wilk test and homogeneity of variance by Levene's test. The effects of alginate type (Zelgan and Neocolloid) and vanillin concentration (control, 0.5%, and 1%) on each outcome were evaluated using two-way analysis of variance (ANOVA). Effect sizes were reported as partial eta-squared (partial η ^2^). For outcomes with a significant main effect of vanillin concentration in the absence of a significant interaction, Tukey's post-hoc test was used for pairwise comparisons. For outcomes that showed a significant alginate × concentration interaction, the effect of vanillin concentration was tested separately within each alginate using Tukey pairwise comparisons. A P value <0.05 was considered statistically significant.

## Results

The physical properties of Zelgan and Neocolloid alginate impression materials incorporated with vanillin at 0%, 0.5% and 1% concentrations were evaluated and are presented in Tables 3.1– 3.6.

**Table 3.1.** Intra-group comparison of physical properties for Zelgan Impression material across Vanillin concentrations (Mean ± SD).

Zelgan				
Parameter	1A (Control) Mean ± SD	1B (0.5% Vanillin) Mean ± SD	1C (1% Vanillin) Mean ± SD	P-value
Gel Strength (MPa)	1.14 ± 0.02	1.06 ± 0.05	1.11 ± 0.06	0.002 *
Permanent Deformation (%)	1.98 ± 0.39	2.14 ± 0.70	2.01 ± 0.46	0.78
Gelation Time (seconds)	100.20 ± 4.05	64.40 ± 2.91	75.60 ± 3.57	<0.001 *
Flow (mm)	44.10 ± 0.88	31.90 ± 0.88	35.60 ± 0.52	<0.001 *
Tear Strength (N/mm)	0.29 ± 0.02	0.29 ± 0.02	0.31 ± 0.05	0.293

* Statistically significant difference from control (P < 0.05), SD = standard deviation

**Table 3.2.** Intra-group comparison of physical properties for Neocolloid impression across vanillin concentrations (Mean ± SD).

Neocolloid				
Parameter	2A (Control) Mean ± SD	2B (0.5% Vanillin) Mean ± SD	2C (1% Vanillin) Mean ± SD	P-value
Gel Strength (MPa)	0.72 ± 0.05	0.69 ± 0.05	0.69 ± 0.03	0.439
Permanent Deformation (%)	1.91 ± 0.72	1.84 ± 0.48	2.01 ± 0.46	0.792
Gelation Time (seconds)	195.60 ± 5.10	175.40 ± 4.35	178.50 ± 3.78	<0.001 *
Flow (mm)	43.80 ± 0.92	38.00 ± 0.82	40.20 ± 0.79	<0.001 *
Tear Strength (N/mm)	0.26 ± 0.02	0.24 ± 0.01	0.28 ± 0.01	<0.001 *

* Statistically significant difference from control (P < 0.05), SD = standard deviation

**Table 3.3.** Inter-group comparison of physical properties for Zelgan & Neocolloid for the control group.

Control- Vanillin 0%					
Parameter	Zelgan (Mean ± SD)		Neocolloid (Mean ± SD)		P-value
	Mean	SD	Mean	SD	
Gel Strength (MPa)	1.14	0.02	0.71	0.05	<0.001 *
Permanent Deformation (%)	1.98	0.39	1.91	0.72	0.779
Gelation Time (seconds)	100.20	4.05	195.60	5.10	<0.001 *
Flow (mm)	44.10	0.88	43.80	0.92	0.497
Tear Strength (N/mm)	0.29	0.02	0.26	0.02	0.014 *

* Statistically significant difference from control (P < 0.05), SD = standard deviation

**Table 3.4.** Inter-group comparison of physical properties for Zelgan & Neocolloid for 0.5% group.

Vanillin 0.5%					
Parameter	Zelgan (Mean ± SD)		Neocolloid (Mean ± SD)		P-value
	Mean	SD	Mean	SD	
Gel Strength (MPa)	1.06	0.05	0.69	0.05	<0.001 *
Permanent Deformation (%)	2.14	0.70	1.84	0.48	0.273
Gelation Time (seconds)	64.40	2.91	175.40	4.35	<0.001 *
Flow (mm)	31.90	0.88	38.00	0.82	<0.001 *
Tear Strength (N/mm)	0.29	0.02	0.24	0.01	<0.001 *

* Statistically significant difference from control (P < 0.05), SD = standard deviation

**Table 3.5.** Inter-group comparison of physical properties for Zelgan & Neocolloid for 1% group.

Vanillin 1%					
Parameter	Zelgan (Mean ± SD)		Neocolloid (Mean ± SD)		P-value
	Mean	SD	Mean	SD	
Gel Strength (MPa)	1.11	0.06	0.69	0.03	<0.001 *
Permanent Deformation (%)	2.01	0.46	2.01	0.46	>0.99
Gelation Time (seconds)	75.60	3.57	178.50	3.78	<0.001 *
Flow (mm)	35.60	0.52	40.20	0.79	<0.001 *
Tear Strength (N/mm)	0.31	0.05	0.28	0.01	0.121

* Statistically significant difference from control (P < 0.05), SD = standard deviation

**Table 3.6.** P-values and partial eta-squared (η ^2^p) values were obtained from a two-way ANOVA evaluating the main effects of alginate type (Zelgan vs. Neocolloid) and concentration (Control, 0.5%, 1%), as well as their interaction, on each outcome measure. η ^2^p = partial eta squared, a measure of effect size.

Outcome	Alginate p (η ^**2**^)	Concentration p (η ^**2**^)	Interaction p (η ^**2**^)
Gel strength	<0.001 (0.957)	0.002 (0.202)	0.080 (0.089)
Permanent deformation	0.380 (0.014)	0.931 (0.003)	0.664 (0.015)
Gelation time	<0.001 (0.995)	<0.001 (0.907)	<0.001 (0.411)
Flow	<0.001 (0.836)	<0.001 (0.960)	<0.001 (0.760)
Tear strength	<0.001 (0.323)	0.004 (0.184)	0.304 (0.043)

Gel Strength: Two-way ANOVA demonstrated significant main effects of alginate type (P < 0.001, partial η ^2^ = 0.957) and vanillin concentration (P = 0.002, partial η ^2^ = 0.202) on gel strength, with no significant alginate × concentration interaction (P = 0.080, partial η ^2^ = 0.089). Tukey post-hoc comparisons showed significantly greater gel strength at the control concentration than at 0.5% vanillin (P = 0.001), while control vs. 1% (P = 0.126) and 0.5% vs. 1% (P = 0.212) were not significant. Inter-group comparison confirmed that Zelgan exhibited consistently and significantly higher gel strength than Neocolloid across all concentrations (P < 0.001).

Permanent Deformation: Two-way ANOVA showed that permanent deformation was not significantly affected by alginate type (P = 0.380, partial η ^2^ = 0.014), vanillin concentration (P = 0.931, partial η ^2^ = 0.003), or their interaction (P = 0.664, partial η ^2^ = 0.015).

Gelation Time: Two-way ANOVA revealed significant main effects of alginate type (P < 0.001, partial η ^2^ = 0.995) and vanillin concentration (P < 0.001, partial η ^2^ = 0.907), together with a significant alginate × concentration interaction (P < 0.001, partial η ^2^ = 0.411), indicating that the effect of vanillin on gelation time differed by material. Tukey pairwise comparisons performed separately within each alginate ( Table 3.1, Table 3.2) showed that all three concentrations differed significantly from one another within Zelgan, whereas in Neocolloid both vanillin concentrations differed significantly from the control but not from each other (P = 0.280). Inter-group comparison confirmed that Zelgan gelled significantly faster than Neocolloid at all concentrations (P < 0.001).

Flow: Two-way ANOVA revealed significant main effects of alginate type (P < 0.001, partial η ^2^ = 0.836) and vanillin concentration (P < 0.001, partial η ^2^ = 0.960), together with a significant alginate × concentration interaction (P < 0.001, partial η ^2^ = 0.760). Tukey pairwise comparisons performed separately within each alginate showed that all three concentrations differed significantly from one another within both materials, with the lowest flow observed at 0.5% vanillin in each case. Inter-group analysis revealed no significant difference at 0% vanillin (P = 0.497), but Neocolloid demonstrated significantly higher flow at 0.5% and 1% concentrations (P < 0.001).

Tear Strength: Two-way ANOVA showed that tear strength was significantly influenced by alginate type (P < 0.001, partial η ^2^ = 0.323) and vanillin concentration (P = 0.004, partial η ^2^ = 0.184), without a significant alginate × concentration interaction (P = 0.304, partial η ^2^ = 0.043), indicating that the effect of vanillin concentration on tear strength did not differ significantly between materials. Tukey post-hoc comparisons showed that tear strength at 1% vanillin was significantly greater than at control (P = 0.019) and 0.5% (P = 0.006); control and 0.5% did not differ (P = 0.911). Inter-group comparison showed Zelgan had significantly higher tear strength than Neocolloid overall (P < 0.001).

## Discussion

The findings of the present study revealed changes in the physical properties of Zelgan and Neocolloid alginate impression materials after incorporating vanillin at concentrations of 0%,0.5%, and 1%. The incorporation of vanillin resulted in noticeable changes in the tested properties of both materials, with some parameters increasing and others decreasing. The magnitude and nature of these changes varied with the type of alginate, indicating that the interaction between vanillin and the alginate matrix is material-dependent.

Disinfection of alginate impression material is commonly performed by spraying or immersion, but because of syneresis and imbibition, these methods are not recommended. Additives like vanillin can be incorporated into alginate impression material, as it is known for its antimicrobial activity and can be an effective way to promote self-disinfection.

Gel strength in the present study decreased after the incorporation of vanillin into both Zelgan and Neocolloid. Any additive capable of influencing the rate or extent of cross-linking is expected to produce corresponding alterations in gel strength. ^15^ The incorporation of vanillin partially replaces alginate in the formulation, thereby reducing the amount of alginate powder available for interaction with a given quantity of water compared to the control group. ^16^ This reduction may decrease the degree of crosslinking, ultimately leading to lower gel strength. Zelgan exhibited consistently higher gel strength than Neocolloid across all concentrations (main effect of alginate type, P < 0.001), which may be attributed to differences in their respective compositions; however, the alginate × concentration interaction was not statistically significant (P = 0.080), indicating that the reduction in gel strength with vanillin did not differ significantly between the two materials.

In the present study, vanillin incorporation significantly reduced gelation time in both alginate impression materials, with the greatest reduction observed at 0.5% concentration. This effect may be attributed to the presence of vanillin particles, which could facilitate localized calcium–alginate crosslinking by providing additional surfaces for interaction, thereby accelerating gel network formation. ^17^ In addition, vanillin may interfere with the action of trisodium phosphate, the retarder component, reducing its effectiveness in delaying calcium alginate formation. The present findings corroborate those of Leelapong et al. (2024), ^18^ who reported a significant reduction in the setting time of alginate impression materials following the incorporation of vanillin at concentrations ranging from 0.1% to 1% (w/w). In agreement with these observations, the present study demonstrated a significant reduction in gelation time after vanillin incorporation. Notably, statistical significance was observed at 0.5% and 1% (w/w) concentrations, indicating that the accelerating effect of vanillin on alginate gelation becomes more pronounced at higher concentrations. This may be attributed to enhanced interactions between vanillin particles and the alginate crosslinking system, leading to accelerated gel formation. Notably, the reduction in Zelgan's gelation time to 64.40 ± 2.91 seconds at 1% vanillin has direct clinical implications: after subtracting the standard 45-second mixing time, only approximately 19 seconds of working time remain to load the tray and seat the impression in the patient's mouth. This falls short of the minimum working time specified under ISO 1563/ADA Specification No. 18 for irreversible hydrocolloid impression materials, indicating that Zelgan modified with 1% vanillin may not be clinically viable at this concentration, whereas Neocolloid maintained an adequate gelation time (175.40-178.50 seconds) across all tested concentrations.

The incorporation of vanillin did not result in a significant alteration in permanent deformation in either Zelgan (P = 0.780) or Neocolloid (P = 0.792), indicating that the elastic recovery of both materials remained unaffected. Since permanent deformation reflects the ability of the calcium alginate gel network to recover following deformation, the present findings suggest that vanillin did not compromise the integrity of the cross-linked polymer matrix. The maintenance of this property implies that the concentration of vanillin incorporated was insufficient to disrupt the network architecture responsible for elastic behaviour. ^19^ From a clinical perspective, the preservation of elastic recovery is desirable, as it ensures accurate reproduction of oral structures, particularly in undercut regions, while minimising the risk of permanent distortion during impression removal. These results therefore demonstrate that vanillin can be incorporated into alginate impression materials without adversely affecting their elastic recovery characteristics.

The present study found that incorporating vanillin did not adversely reduce the tear strength of either alginate material, indicating that the additive did not compromise the structural integrity of the calcium alginate gel network. For Zelgan specifically, this suggests that the incorporated vanillin concentration was insufficient to disrupt the intermolecular interactions responsible for resistance to tensile stress. ^20^

Two-way ANOVA showed that vanillin concentration significantly increased tear strength at 1% compared with control and 0.5% (P = 0.019 and P = 0.006, respectively), with no significant alginate × concentration interaction (P = 0.304). This indicates that the increase in tear strength at 1% vanillin was a general effect of concentration that did not differ significantly between Zelgan and Neocolloid, rather than a property specific to one material. As no chemical or microstructural characterisation (e.g., FTIR, SEM) was performed in this study, the following explanation should be regarded as a hypothesis rather than a confirmed mechanism: the observed improvement in tear strength may be attributed to the uniform dispersion of vanillin particles within the alginate matrix, where they could potentially act as reinforcing micro-fillers. The incorporation of such particulate additives may enhance the structural cohesion of the gel network by facilitating a more homogeneous distribution of applied stresses throughout the cross-linked matrix. Consequently, the material may exhibit increased resistance to crack initiation and propagation under tensile loading. In addition, vanillin particles may promote physical interactions with the surrounding polymer network, thereby improving filler–matrix interfacial adhesion and contributing to localised reinforcement of the calcium alginate structure. These proposed effects would need to be confirmed through chemical or microstructural analysis in future work, but may collectively enhance the mechanical integrity of the gel, thereby improving its resistance to tearing. ^20^

From a clinical perspective, preserving or enhancing tear strength is advantageous, as it reduces the likelihood of impression tearing during removal, thereby improving the accuracy and completeness of impressions obtained from regions with deep embrasures or undercut anatomical structures. These findings indicate that vanillin incorporation, particularly at a concentration of 1%, may provide additional reinforcement to the alginate matrix without compromising its mechanical performance. Although the present study’s findings suggest that vanillin can be incorporated into alginate impression materials without adversely affecting key physical properties, additional investigations are needed to comprehensively evaluate its effects on other clinically relevant characteristics. In particular, further studies should assess the effects of vanillin incorporation on detail reproduction and dimensional stability, as these properties are critical determinants of impression accuracy and the subsequent fabrication of precise dental casts. Such investigations would provide a more comprehensive understanding of the suitability of vanillin as an antimicrobial additive in irreversible hydrocolloid impression materials.

## Conclusion

The incorporation of vanillin at concentrations of 0.5% and 1.0% modified the measured physicochemical properties of the alginate impression materials. Two-way ANOVA confirmed that the effect of vanillin concentration was material-dependent (significant alginate × concentration interaction) for gelation time and flow, whereas its effects on gel strength and tear strength did not differ significantly between the two materials. For Neocolloid, the observed changes in gel strength, permanent deformation, gelation time, flow, and tear strength remained within the ranges tested, with gelation time remaining above 175 seconds across all tested concentrations, supporting its potential as a viable antimicrobial additive. For Zelgan, however, 1% vanillin reduced gelation time to 64.40 ± 2.91 seconds, leaving an estimated working time of only ~19 seconds after mixing — a reduction that raises concern regarding clinical viability at this concentration. These conclusions are limited to the five in vitro properties measured in this study; working time, dimensional stability, detail reproduction, and compliance with ISO 21563:2021 were not directly evaluated, and the antimicrobial efficacy of the vanillin-incorporated materials was not assessed. These findings indicate that vanillin may be a suitable additive for Neocolloid at the concentrations tested, but further studies assessing the properties above are needed before its clinical suitability, including for Zelgan, can be established.

## Data Availability

Figshare: Tables, Mean & standard deviation values. https://doi.org/10.6084/m9.figshare.32660919. ^21^ This project contains the following data:
• Tables.docx • THESIS RESULTS • 2-WAY ANOVA Data are available under the terms of the Creative Commons Zero "No rights reserved" data waiver (CC0 1.0 Public domain dedication).
