# Supplementary material for: Influence of Vanillin at Different Concentrations on the Properties of Commercial Alginate Impression Materials: An in-vitro Study
Source: F1000Res. 2026 Oct 6;15:1224. Originally published 2026 Jul 27. [Version 2] doi: 10.12688/f1000research.184245.2 (PMC13722854; doi:10.12688/f1000research.184245.2)
Supplement: Peer review history

# Reviewer report: Reviewer response for version 2

**DOI:** 10.5256/f1000research.211446.r527741

**Published:** 2026-10-30

**Authors:** Rene Garcia-Contreras

**Affiliations:** 1 ENES León, Nanostructures and Biomaterials Area, National Autonomous University of Mexico (UNAM), Leon, Guanajuato, Mexico

Thank you for inviting me to reassess the revised manuscript entitled “Influence of Vanillin at Different Concentrations on the Properties of Commercial Alginate Impression Materials: An In Vitro Study.”

 I have reviewed Version 2 and the authors' responses to my previous comments. The manuscript has been substantially improved, particularly regarding the methodology, statistical analysis, data presentation, and interpretation of the results.

 I appreciate the authors' efforts in addressing my suggestions. Although some minor limitations remain, I consider that the revised manuscript meets an acceptable scientific standard.

 I am pleased to change my previous recommendation from “Approved with reservations” to “Approved.”

 The manuscript is accepted in its present form.

 Best regards,

 René García-Contreras

Is the work clearly and accurately presented and does it cite the current literature?

Partly

If applicable, is the statistical analysis and its interpretation appropriate?

Partly

Are all the source data underlying the results available to ensure full reproducibility?

Partly

Is the study design appropriate and is the work technically sound?

Partly

Are the conclusions drawn adequately supported by the results?

No

Are sufficient details of methods and analysis provided to allow replication by others?

No

Reviewer Expertise:

Dental biomaterials, alginate impression materials, antimicrobial materials, biocompatibility, nanobiotechnology, tissue engineering, and in vitro evaluation of dental materials.

I confirm that I have read this submission and believe that I have an appropriate level of expertise to confirm that it is of an acceptable scientific standard.

# Reviewer report: Reviewer response for version 2

**DOI:** 10.5256/f1000research.211446.r527742

**Published:** 2026-10-15

**Authors:** Pankaj Dhawan

**Affiliations:** 1 Manav Rachna International Institute Of Research And Studies, Faridabad, Haryana, India

1. While Version 2 successfully incorporated vital parameters such as water temperature (20^\circ\text{C}) and powder-to-water ratios (Zelgan 18g:36mL; Neocolloid 18g:36mL), the study design still lacks explicit reporting on ambient room temperature and relative humidity during mixing and testing. Because irreversible hydrocolloids are highly sensitive to climatic fluctuations, omitting ambient environmental controls remains a minor reproducibility vulnerability.

 2. The introduction states that vanillin was previously investigated at *0.1%, 0.5%, and 1% w/w*, but the current study's methodology and testing matrix (Table 2) evaluate only *control, 0.5%, and 1%*. To avoid reader confusion regarding the scope, the introduction should explicitly clarify that while prior literature tested 0.1%, the present study focuses exclusively on 0.5% and 1% to isolate clinically viable thresholds.

 3. The discussion attributes the increase in tear strength at 1% vanillin to particles acting as reinforcing micro-fillers. Because no microstructural or chemical characterization (such as Scanning Electron Microscopy [SEM] or Fourier-Transform Infrared Spectroscopy [FTIR]) was performed, the authors correctly frame this as a hypothesis. *The authors should clearly distinguish between hypothesized and experimentally verified chemical cross-linking to ensure that readers do not interpret speculation as confirmed evidence.*

 4. The manuscript has undergone substantial, rigorous improvement following peer-review feedback. With the explicit limitation that **antimicrobial efficacy, dimensional stability, detail reproduction, and exact clinical viability for Zelgan at 1% require future investigation**, the paper represents a sound, methodologically rigorous in vitro contribution to dental biomaterials literature.

*The article may be considered approved with the highlighted changes.*

Is the work clearly and accurately presented and does it cite the current literature?

No

If applicable, is the statistical analysis and its interpretation appropriate?

Partly

Are all the source data underlying the results available to ensure full reproducibility?

Partly

Is the study design appropriate and is the work technically sound?

No

Are the conclusions drawn adequately supported by the results?

No

Are sufficient details of methods and analysis provided to allow replication by others?

No

Reviewer Expertise:

Prosthodontics, Dental Materials.

I confirm that I have read this submission and believe that I have an appropriate level of expertise to confirm that it is of an acceptable scientific standard.

# Reviewer report: Reviewer response for version 1

**DOI:** 10.5256/f1000research.203372.r519146

**Published:** 2026-09-11

**Authors:** Rene Garcia-Contreras

**Affiliations:** 1 ENES León, Nanostructures and Biomaterials Area, National Autonomous University of Mexico (UNAM), Leon, Guanajuato, Mexico

This in vitro study evaluates the influence of 0.5% and 1% vanillin on the physical properties of two commercial alginate impression materials. The topic is relevant, and the comparison between Zelgan and Neocolloid provides potentially useful information. However, several issues should be addressed to strengthen the reliability and clinical interpretation of the findings.

**Introduction**

 The novelty should be clarified because Reference 18 previously evaluated similar vanillin concentrations. The authors could emphasize the comparison between two commercial materials and the assessment of additional physical properties. The current literature, particularly ISO 21563:2021, should also be incorporated.

**Methods**

 Please provide the exact powder-to-water ratios, water and room temperatures, product batch numbers, number of independent mixtures, specimens obtained per mixture, and instrument calibration procedures. It should also be clarified whether each specimen represents an independent experimental unit.

 The discrepancy between the one-minute mixing time reported in the abstract and the 45 seconds stated in the Methods should be corrected.

**Statistical analysis**

 Because the study follows a 2 × 3 factorial designs, a two-way analysis including material, vanillin concentration, and their interaction would better support the proposed material-dependent effect. Please specify the test used for each outcome and report adjusted post-hoc comparisons, effect sizes, and confidence intervals.

**Source data**

 The availability of individual data is appreciated. Nevertheless, the dataset should be carefully verified because:

- The Neocolloid 1% permanent-deformation values are identical to those reported for Zelgan 1%.
- One tear-strength value is entered as “0.26.5.”
- Neocolloid control gel strength is reported as both 0.71 and 0.72 MPa.

Correcting these points and uploading an updated dataset would improve reproducibility.

**Results and discussion**

 Some concentration-specific conclusions are based only on omnibus P values. Please provide the corresponding post-hoc results. The discussion of tear strength should also be revised because it first indicates no significant effect and later describes a significant increase in Neocolloid.

 The proposed cross-linking and reinforcement mechanisms are interesting, but they should be presented as hypotheses because no chemical or microstructural characterization was performed.

**Conclusions**

 The statement that the changes remained within clinically acceptable limits is not sufficiently supported because working time, dimensional stability, detail reproduction, and compliance with ISO 21563:2021 were not directly evaluated. Antimicrobial efficacy was also not assessed in the tested materials.

 I encourage the authors to limit the conclusions to the measured in vitro properties and to revise the manuscript after verifying the dataset and reanalyzing the factorial design.

Is the work clearly and accurately presented and does it cite the current literature?

Partly

If applicable, is the statistical analysis and its interpretation appropriate?

Partly

Are all the source data underlying the results available to ensure full reproducibility?

Partly

Is the study design appropriate and is the work technically sound?

Partly

Are the conclusions drawn adequately supported by the results?

No

Are sufficient details of methods and analysis provided to allow replication by others?

No

Reviewer Expertise:

Dental biomaterials, alginate impression materials, antimicrobial materials, biocompatibility, nanobiotechnology, tissue engineering, and in vitro evaluation of dental materials.

I confirm that I have read this submission and believe that I have an appropriate level of expertise to confirm that it is of an acceptable scientific standard, however I have significant reservations, as outlined above.

# Response

**Published:** 2026-09-25

**Authors:** Nayana Prabhu

**Affiliations:** Prosthodontics, Manipal College of Dental Sciences, Manipal, Karnataka, India

**Reviewer Comment:** *This in vitro study evaluates the influence of 0.5% and 1% vanillin on the physical properties of two commercial alginate impression materials. The topic is relevant, and the comparison between Zelgan and Neocolloid provides potentially useful information. However, several issues should be addressed to strengthen the reliability and clinical interpretation of the findings.*

**Author Response: **We thank the reviewer for the positive assessment of the study's relevance and for the constructive comments below, all of which we have addressed.

**Reviewer Comment:** *The novelty should be clarified because Reference 18 previously evaluated similar vanillin concentrations. The authors could emphasize the comparison between two commercial materials and the assessment of additional physical properties. The current literature, particularly ISO 21563:2021, should also be incorporated.*

**Author Response: **We agree. The Introduction has been revised to explicitly acknowledge that Leelapong et al. (2024, Reference 18) previously evaluated similar vanillin concentrations (0.1-1% w/w) in a single commercial product under ISO 21563:2021, assessing working time, setting time, elastic recovery, and strain in compression. We have clarified that the novelty of the present study lies in the direct comparison of two distinct commercial alginate formulations and the assessment of additional physical properties (gel strength, permanent deformation, tear strength) not evaluated in that prior work. The ISO 21563:2021 standard is now cited in this context.

**Reviewer Comment:** *Please provide the exact powder-to-water ratios, water and room temperatures, product batch numbers, number of independent mixtures, specimens obtained per mixture, and instrument calibration procedures. It should also be clarified whether each specimen represents an independent experimental unit.*

**Author Response: **We agree. The Specimen Preparation subsection now specifies the water temperature, powder-to-water ratio for each material, product batch numbers, number of independent mixtures per group, number of specimens obtained per mixture, and the calibration procedure for the testing instruments; specimen independence is also clarified.

**Reviewer Comment:** *The discrepancy between the one-minute mixing time reported in the abstract and the 45 seconds stated in the Methods should be corrected.*

**Author Response: **Corrected. The Abstract now states a 45-second mixing time, consistent with the Methods section.

**Reviewer Comment:** *Because the study follows a 2 × 3 factorial designs, a two-way analysis including material, vanillin concentration, and their interaction would better support the proposed material-dependent effect. Please specify the test used for each outcome and report adjusted post-hoc comparisons, effect sizes, and confidence intervals.*

**Author Response: **We agree, and this analysis has now been performed. A two-way ANOVA (alginate type × vanillin concentration) was conducted for each of the five outcomes; results (main effects, interaction, and partial eta-squared effect sizes) are reported in the Results text and summarised in a new Table 3.6. Where the interaction was significant (gelation time, flow), Tukey pairwise comparisons were performed separately within each alginate, and the resulting significance groupings are indicated with superscript letters in Tables 3.1 and 3.2. Where the interaction was not significant (gel strength, tear strength, permanent deformation), Tukey post-hoc comparisons were performed on the pooled concentration main effect. The Statistical Analysis subsection of the Methods has been rewritten to describe this approach, including Levene's test for homogeneity of variance. Exact P-values and effect sizes are now reported for every comparison; 95% confidence intervals can be added for the Tukey comparisons if the reviewer considers this necessary in addition to the exact P-values and effect sizes already provided.

**Reviewer Comment:** *The availability of individual data is appreciated. Nevertheless, the dataset should be carefully verified because: (1) the Neocolloid 1% permanent-deformation values are identical to those reported for Zelgan 1%; (2) one tear-strength value is entered as "0.26.5"; (3) Neocolloid control gel strength is reported as both 0.71 and 0.72 MPa. Correcting these points and uploading an updated dataset would improve reproducibility.*

**Author Response: **We thank the reviewer for this careful check. Regarding point (3), we confirm this was a manuscript error: the raw dataset mean for Neocolloid control gel strength is 0.711 MPa (rounding to 0.71), matching Table 3.3; Table 3.2 has been corrected from 0.72 to 0.71 MPa. Regarding point (2), we traced the "0.26.5" entry in the source spreadsheet; because spreadsheet software ignores non-numeric text when computing an average, this entry did not affect the reported mean tear strength (recomputed from the nine valid values, mean = 0.258, matching the value already reported), but we agree the raw cell itself requires correction and will fix it to 0.265 before re-uploading the dataset. Regarding point (1), we have confirmed that the Neocolloid 1% permanent-deformation column in the source spreadsheet is indeed identical to the Zelgan 1% column; this reflects a data-entry error during compilation.

**Reviewer Comment:** *Some concentration-specific conclusions are based only on omnibus P values. Please provide the corresponding post-hoc results.*

**Author Response: **Addressed as part of the two-way ANOVA revision above: pairwise Tukey post-hoc comparisons (with exact P-values) are now reported in the Results text for every outcome, rather than the omnibus ANOVA P-value alone.

**Reviewer Comment:** *The discussion of tear strength should also be revised because it first indicates no significant effect and later describes a significant increase in Neocolloid.*

**Author Response: **We agree and have corrected this. Re-analysis by two-way ANOVA showed that tear strength was significantly increased at 1% vanillin compared with control and 0.5% (P=0.019 and P=0.006), with no significant alginate × concentration interaction (P=0.304) - i.e., this increase is a general concentration effect that does not differ significantly between Zelgan and Neocolloid, rather than a Neocolloid-specific effect. Both the Results and Discussion sections have been rewritten to remove the internal contradiction and present this single, statistically consistent interpretation.

**Reviewer Comment:** *The proposed cross-linking and reinforcement mechanisms are interesting, but they should be presented as hypotheses because no chemical or microstructural characterization was performed.*

**Author Response: **Agreed. The relevant Discussion paragraph now explicitly states that, as no chemical or microstructural characterisation (e.g., FTIR, SEM) was performed in this study, the proposed micro-filler/reinforcement mechanism should be regarded as a hypothesis rather than a confirmed mechanism, and that it would need to be verified by such analyses in future work.

**Reviewer Comment:** *The statement that the changes remained within clinically acceptable limits is not sufficiently supported because working time, dimensional stability, detail reproduction, and compliance with ISO 21563:2021 were not directly evaluated. Antimicrobial efficacy was also not assessed in the tested materials.*

**Author Response: **We agree and have revised the Conclusion accordingly. It now explicitly states that the conclusions are limited to the five in vitro properties actually measured (gel strength, permanent deformation, gelation time, flow, tear strength), and that working time, dimensional stability, detail reproduction, compliance with ISO 21563:2021, and antimicrobial efficacy were not directly evaluated in this study and require further investigation.

**Reviewer Comment:** *I encourage the authors to limit the conclusions to the measured in vitro properties and to revise the manuscript after verifying the dataset and reanalyzing the factorial design.*

**Author Response: **We thank the reviewer for this guidance. The Conclusion, Abstract, Results, and Discussion have been revised to reflect only the measured in vitro properties, framed within the appropriate two-way factorial analysis.

# Reviewer report: Reviewer response for version 1

**DOI:** 10.5256/f1000research.203372.r516382

**Published:** 2026-09-04

**Authors:** Pankaj Dhawan

**Affiliations:** 1 Manav Rachna International Institute Of Research And Studies, Faridabad, Haryana, India

1.  The methodology omits the specific water temperature used during mixing. Because alginate gelation is highly sensitive to temperature variations, this omission prevents accurate replication of the study.
2.  The authors conclude that the observed changes remain "within clinically acceptable limits". However, Zelgan's gelation time dropped to **64.4 seconds**. Subtracting the **45-second** mixing time leaves only ~19 seconds of working time to load the tray and seat it in the patient's mouth, which is highly unlikely to be clinically acceptable.
3.  In the Introduction, rewrite "Irreversible hydrocolloid impression materials, widely known as alginates, are widely used..." to "Irreversible hydrocolloid impression materials, commonly known as alginates, are widely used..." to remove repetitive phrasing and correct the capitalization.
4.  The authors mention using an effect size of **0.63** for their *a priori* sample size estimation. However, they fail to report the actual measured effect sizes (e.g., Cohen's) for their experimental results to validate the clinical significance of the findings.
5.  Essential variables required for reproducing the study are missing. The authors do not state the exact water temperature used, nor do they specify the exact powder-to-water ratios, relying vaguely on "the respective manufacturer's instructions". Because alginate setting times are highly sensitive to water temperature, this is a major gap.
6.  To scientifically justify the claim that the modified materials perform "within clinically acceptable limits", the authors must cite the specific ISO standard (e.g., ISO 21563 for alginate impression materials) or ADA specifications that outline the minimum acceptable working and setting times. Without this citation, their conclusion lacks a factual basis.
7.  The reference list contains some formatting anomalies, such as a lack of spaces after the publication year and volume in several references (e.g., references 17, 18, and 19). 
8.  The conclusion that vanillin alters properties in a "material-dependent manner" is well-supported. For example, the results show that vanillin significantly decreased the gel strength of Zelgan but had no significant effect on Neocolloid. Similarly, tear strength increased significantly for Neocolloid at 1% vanillin but remained unchanged for Zelgan.
9.  In the main text, in conclusion, the authors claim, "the magnitude of these changes remained within clinically acceptable limits, indicating that vanillin incorporation does not adversely affect the functional performance of the materials." This statement is directly contradicted by their data **.**
10. **Explicitly** state that while vanillin might be a viable additive for Neocolloid (which maintained a 175+ second gelation time), it severely compromises the working time of Zelgan, rendering it clinically unviable at the tested concentrations.

Is the work clearly and accurately presented and does it cite the current literature?

No

If applicable, is the statistical analysis and its interpretation appropriate?

Partly

Are all the source data underlying the results available to ensure full reproducibility?

Partly

Is the study design appropriate and is the work technically sound?

No

Are the conclusions drawn adequately supported by the results?

No

Are sufficient details of methods and analysis provided to allow replication by others?

No

Reviewer Expertise:

Prosthodontics, Dental Materials.

I confirm that I have read this submission and believe that I have an appropriate level of expertise to state that I do not consider it to be of an acceptable scientific standard, for reasons outlined above.

# Response

**Published:** 2026-09-25

**Authors:** Nayana Prabhu

**Affiliations:** Prosthodontics, Manipal College of Dental Sciences, Manipal, Karnataka, India

**Reviewer Comment:** *The methodology omits the specific water temperature used during mixing. Because alginate gelation is highly sensitive to temperature variations, this omission prevents accurate replication of the study.*

**Author Response: **We agree. We have added a statement specifying the water temperature used during mixing to the Specimen Preparation subsection of the Methods.

**Reviewer Comment:** *The authors conclude that the observed changes remain "within clinically acceptable limits". However, Zelgan's gelation time dropped to 64.4 seconds. Subtracting the 45-second mixing time leaves only ~19 seconds of working time to load the tray and seat it in the patient's mouth, which is highly unlikely to be clinically acceptable.*

**Author Response: **We agree with this observation and have revised both the Discussion and Conclusion. A new paragraph has been added to the Discussion explicitly stating that the reduction in Zelgan's gelation time to 64.40 ± 2.91 seconds at 1% vanillin leaves an estimated working time of only ~19 seconds after the 45-second mixing period, which does not meet the minimum working time specified under ISO 1563 / ADA Specification No. 18. The Conclusion has been revised accordingly to state that this raises concern regarding the clinical viability of Zelgan at this concentration, while Neocolloid maintained an adequate gelation time (175.40-178.50 seconds) throughout.

**Reviewer Comment:** *In the Introduction, rewrite "Irreversible hydrocolloid impression materials, widely known as alginates, are widely used..." to "Irreversible hydrocolloid impression materials, commonly known as alginates, are widely used..." to remove repetitive phrasing and correct the capitalization.*

**Author Response: **Done. The sentence has been revised exactly as suggested in the Introduction, and the capitalisation of "alginates" has been corrected to lower case.

**Reviewer Comment:** *The authors mention using an effect size of 0.63 for their a priori sample size estimation. However, they fail to report the actual measured effect sizes (e.g., Cohen's) for their experimental results to validate the clinical significance of the findings.*

**Author Response: **We agree. Following two-way ANOVA of all outcomes (see response below), we now report partial eta-squared (partial η²) effect sizes for the main effects of alginate type, vanillin concentration, and their interaction for every outcome in a new summary table (Table 3.6). A sentence has also been added to the Methods (sample-size paragraph) noting that measured effect sizes are reported alongside P-values in the Results section.

**Reviewer Comment:** *Essential variables required for reproducing the study are missing. The authors do not state the exact water temperature used, nor do they specify the exact powder-to-water ratios, relying vaguely on "the respective manufacturer's instructions". Because alginate setting times are highly sensitive to water temperature, this is a major gap.*

**Author Response: **We agree. The Specimen Preparation subsection has been revised to include the water temperature, the powder-to-water ratio for each material, product batch numbers, the number of independent mixtures prepared per group, the number of specimens obtained per mixture, and the instrument calibration procedure.

**Reviewer Comment:** *To scientifically justify the claim that the modified materials perform "within clinically acceptable limits", the authors must cite the specific ISO standard (e.g., ISO 21563 for alginate impression materials) or ADA specifications that outline the minimum acceptable working and setting times. Without this citation, their conclusion lacks a factual basis.*

**Author Response: **We agree. ISO 1563 / ADA Specification No. 18 (the specification governing minimum working and setting times for irreversible hydrocolloid impression materials) is now explicitly cited in the Discussion and Conclusion wherever a claim of "clinically acceptable limits" is made, replacing the previously uncited statement.

**Reviewer Comment:** *The reference list contains some formatting anomalies, such as a lack of spaces after the publication year and volume in several references (e.g., references 17, 18, and 19).*

**Author Response: **Reference 17 contained a missing space after a full stop ("vanillin.Experiments"), which has been corrected to "vanillin. Experiments".

**Reviewer Comment:** *The conclusion that vanillin alters properties in a "material-dependent manner" is well-supported. For example, the results show that vanillin significantly decreased the gel strength of Zelgan but had no significant effect on Neocolloid. Similarly, tear strength increased significantly for Neocolloid at 1% vanillin but remained unchanged for Zelgan.*

**Author Response: **We thank the reviewer for this observation. In response to a related request from another reviewer for a formal two-way ANOVA (alginate type × vanillin concentration), we re-analysed all five outcomes. This factorial analysis showed that gelation time and flow have a statistically significant alginate × concentration interaction (P<0.001 for both), confirming a genuine material-dependent effect for these two properties. However, for gel strength (interaction P=0.080) and tear strength (interaction P=0.304), the interaction was not statistically significant: both properties were influenced by vanillin concentration and differed between materials as main effects, but the *effect of concentration on these two properties did not differ significantly between Zelgan and Neocolloid*. We have therefore revised the Results, Discussion, and Conclusion to state that the material-dependent (interactive) effect of vanillin applies specifically to gelation time and flow, while its effects on gel strength and tear strength are general across both materials. We believe this more precise, statistically supported description is preferable to the original framing, and we thank the reviewer for prompting this deeper analysis.

**Reviewer Comment:** *In the main text, in conclusion, the authors claim, "the magnitude of these changes remained within clinically acceptable limits, indicating that vanillin incorporation does not adversely affect the functional performance of the materials." This statement is directly contradicted by their data.*

**Author Response: **We agree and have removed this blanket statement. The Conclusion has been rewritten to differentiate the two materials explicitly (see also response to the next comment) rather than making a single unqualified claim for both.

**Reviewer Comment:** *Explicitly state that while vanillin might be a viable additive for Neocolloid (which maintained a 175+ second gelation time), it severely compromises the working time of Zelgan, rendering it clinically unviable at the tested concentrations.*

**Author Response: **Done. The Conclusion now explicitly states that vanillin may be a suitable additive for Neocolloid at the concentrations tested (gelation time remained above 175 seconds throughout), whereas for Zelgan, 1% vanillin reduced gelation time to 64.40 ± 2.91 seconds, leaving only ~19 seconds of estimated working time — a reduction that raises clear concern regarding its clinical viability at this concentration. The Abstract's Conclusions section has been updated to remain consistent with this revised, material-specific conclusion.
